# Isolated vertebral fractures give elevated serum protein S-100B levels

**DOI:** 10.1186/1757-7241-16-13

**Published:** 2008-11-07

**Authors:** Lorin M Benneker, Christoph Leitner, Luca Martinolli, Kretschmer Robert, Heinz Zimmermann, Aristomenis K Exadaktylos

**Affiliations:** 1 Department of Emergency Medicine, Bern University Hospital, Switzerland; 2 Department of Orthopaedic and Trauma Surgery, Bern University Hospital, Switzerland; 3 Department of Analytical Chemistry, Bern University Hospital Switzerland

## Abstract

**Background:**

Serum protein S-100B determinations have been widely proposed in the past as markers of traumatic brain injury and used as a predictor of injury severity and outcome. The purpose of this prospective observational case series was therefore to determine S-100B serum levels in patients with isolated injuries to the back.

**Methods:**

Between 1 February and 1 May 2008, serum samples for S-100B analysis were obtained within 1 hour of injury from 285 trauma patients. All patients with a head injury, polytrauma, and intoxicated patients were excluded to select isolated injuries to the spine. 19 patients with isolated injury of the back were included. Serum samples for S-100B analysis and CT spine were obtained within 1 hours of injury.

**Results:**

CT scans showed vertebral fractures in 12 of the 19 patients (63%). All patients with fractures had elevated S-100B levels. Amongst the remaining 7 patients without a fracture, only one patient with a severe spinal contusion had an S-100B concentration above the reference limit. The mean S-100B value of the group with fractures was more than 4 times higher than in the group without fractures (0.385 vs 0.087 μg/L, p = 0.0097).

**Conclusion:**

Our data, although limited due to a very small sample size, suggest that S-100B serum levels might be useful for the diagnosis of acute vertebral body and spinal cord injury with a high negative predictive power. According to the literature, the highest levels of serum S-100B are found when large bones are fractured. If a large prospective study confirms our findings, determining the S-100B level may contribute to more selective use of CT and MRI in spinal trauma.

## Background

The reliable detection of spinal injury remains difficult despite major progress in imaging techniques. Computed tomography and now MRI are recommended as the gold standard for the early detection of spinal injuries. The indication for computed tomography in spinal trauma patients is still controversial, as not all hospitals have 24-hour access to such modalities and transfers are cost-intensive and may be linked to increased morbidity and mortality. Serum protein S-100B determinations have been widely proposed in the past as markers of traumatic brain injury and used as a predictor of injury severity and outcome. [1-4] S-100 protein is a low molecular weight protein found in vertebrates characterized by two calcium binding sites of the helix-loop-helix ("EF-hand type") conformation. There are at least 21 different types of S-100 protein. The name is derived from the fact that the protein is 100% soluble in ammonium sulfate at neutral pH. S-100 is normally present in cells derived from the neural crest (Schwann cells, melanocytes, and glial cells), chondrocytes, adipocytes, myoepithelial cells, macrophages, Langerhans cells, dendritic cells, and keratinocytes. S-100 proteins have been implicated in a variety of intracellular and extracellular functions. S100 proteins are involved in regulation of protein phosphorylation, transcription factors, Ca++ homeostasis, the dynamics of cytoskeleton constituents, enzyme activities, cell growth and differentiation, and the inflammatory response.[5] Recent studies suggested that S-100B protein may be a biomarker for traumatic spinal cord injury in an animal model [6]. In trauma patients, S-100B shows a high negative predictive power for head injuries, but is also elevated in larger extracranial injuries, especially in fractures. [7,8] However, little is known about the effects of isolated vertebral injuries on S-100B serum concentrations.

The purpose of this prospective observational case series was therefore to determine the levels of S-100B serum levels in patients with isolated injuries to the spine. Our aim was to determine whether S-100 B is a reliable serum marker for spinal injuries and, if so, whether it is reliable in spinal injury both with and without fractures of the spinal column.

## Methods

Our site is a Swiss level-one emergency unit with a 24-hour trauma service, seeing about 500 severely injured (ISS > 16) patients per year.

Between 1 February and 1 May 2008, serum samples for S-100B analysis were obtained within 1 hour of injury from285 trauma patients. All patients with a head injury, GCS < 15, polytrauma, patients with limb or torso injuries, intoxicated or sedated patients, and patients with an unreliable history were excluded to select isolated injuries to the spine. 19 patients with isolated injuries to the spine remained for further analysis. The samples were assessed using a S-100 ECLIA (electrochemiluminescence immunoassay) method on a Modular Analytics E170 automated analyser (Roche Diagnostics AG, Rotkreuz, Switzerland). S-100B serum were designated as positive if concentrations were higher than 0.10 μg/L, the upper 95% reference limit for a normal healthy population. [7]

CT scans of the head and the complete spine from all patients were obtained using a 16-row detector CT scanner (Sensation16, Siemens, Erlangen, Germany) with a collimation between 0.75 and 1.5 mm. The scans were evaluated for traumatic lesions independently by a neuroradiologist and a spine surgeon who were blinded to the results of the S-100B measures.

## Results

CT scans showed vertebral fractures in 12 of the 19 patients (63%). All patients with fractures had elevated S-100B levels. Amongst the remaining 7 patients without a fracture, only one patient with a severe spinal contusion had an S-100B concentration above the reference limit. 4 of the 19 patients showed neurological symptoms; all these patients has clearly elevated S100B concentrations and all but the above mentioned patient with a spinal contusion had instable fractures (Table 1). The mean S-100B value in the group with fractures was more than 4 times higher than in the group without fractures (0.385 vs 0.087 μg/l, p = 0.0097). No correlation was found between age, gender or location of the fracture. Overall it seems that fractures of larger vertebrae and more complex fractures result in higher S-100B values. (Table 1)

**Table 1:** Patient characteristics, S 100B levels and diagnosis

**No**	**gender**	**age**	**S100B ug/L**	**type of injury**
1	f	59	0.046	strain trauma, no fracture
2	m	31	0.658	compression fracture T6, T11 (Type A 1.2)
3	f	21	0.238	burst fracture C7 (Type A 3.1)
4	m	30	0.225	bullet through massa lateralis C1 and spinal cord, no neurological symptoms
5	m	17	0.115	compression fracture T7- 9 (A 1.1)
6	m	40	0.226	burst fracture L1 (A 3.1)
7	m	28	0.067	strain trauma, no fracture
8	m	32	0.072	strain trauma, no fracture
9	m	75	0.253	compression fractures L4, L5 (A 1,2)
10	m	30	0.108	inferior articular process and arcus fracture C6
11	m	38	0.715	flexion/distraction fracture T5/6 (B 2.3), paraplegia
12	m	44	0.063	distortion, no fracture
13	m	84	0.201	dense fracture type 2, Jefferson fracture C1
14	m	63	0.221	contusio spinalis C3-5, transient tetraplegia
15	m	68	0.481	subluxation C5/6 with fracture C6 (B 1.2.1)
16	m	22	0.048	strain trauma, no fracture
17	m	44	0.921	burst-split fracture L1 (A 3.3), transient hyposthesia
18	f	60	0.483	luxation fracture C6-7, radiculopathy C6
19	f	17	0.096	thoracal vertebral contusion, no fracture

## Discussion

S100B has a high positive and negative predictive power, and the finding of a normal S100B value shortly after trauma may exclude significant injury. Our data, although limited due to a very small sample size, suggest that S-100B serum levels might be a useful tool for the diagnosis of acute vertebral body and spinal cord injury. According to the literature, the highest levels of serum S-100B are found when large bones are fractured [7]. The clear correlation between the vertebral fractures and elevated S-100B serum concentrations and the tendency to higher values with more complex fractures of larger bones make the bone marrow a more likely source of S-100B in serum than potentially injured nerve tissue. However, spinal cord injuries without fractures can also apparently elevate S-100B serum concentrations, although our sample was too small for reliable conclusions or statistics. If a large prospective study confirms our findings, determining the S-100B level may contribute to more selective use of CT and MRI in spinal trauma. It should be borne in mind that positive S-100B results may be over interpreted as a reliable positive predictor for injuries to the spine, especially if further injuries are present. In our sample, however, normal S-100B concentration were not found in the presence of relevant injuries. A limitation of our study is that axonal injuries cannot be excluded, even in patients without history of percussive trauma to the head. Such patients may show elevated S-100B levels as may patients with large soft-tissue injuries, although our aim was to exclude such cases we tried to exclude such as far as possible [1,7].

## Conclusion

The clinical impact of our positive S-100B findings is limited. It can, however, be assumed that patients with reduced consciousness (intoxicated, intubated, sedated) and a negative S-100B have no significant brain or spinal injury. This might contribute to sparing patients from unnecessary exposure to radiation, saving resources and ultimately reducing costs. Although our results are promising, no firm conclusions can be drawn at present. Future efforts to develop biomarkers that predict functional outcomes in the acute phase of spinal injury should also focus on axon-specific proteins.[8]

## Competing interests

The authors declare that they have no competing interests.

## Authors' contributions

LMB was responsible for data analysis and writing of the manuscript. CL and LM were collecting data. RK conducted all biochemical assessments. HZ performed the statistical analysis. AKE was responsible for study design and writing of the manuscript. All authors read and approved the final manuscript.
